# A synthetic digital city dataset for robustness and generalisation of depth estimation models

**DOI:** 10.1038/s41597-024-03025-5

**Published:** 2024-03-16

**Authors:** Jihao Li, Jincheng Hu, Yanjun Huang, Zheng Chen, Bingzhao Gao, Jingjing Jiang, Yuanjian Zhang

**Affiliations:** 1 Department of Aeronautical and Automotive Engineering, Loughborough University, Leicestershire, LE11 3TU UK; 2 School of Automotive Studies, Tongji University, ShangHai, 201804 China; 3 Faculty of Transportation Engineering, Kunming University of Science and Technology, 650500 Kunming, China

**Keywords:** Electrical and electronic engineering, Computer science

## Abstract

Existing monocular depth estimation driving datasets are limited in the number of images and the diversity of driving conditions. The images of datasets are commonly in a low resolution and the depth maps are sparse. To overcome these limitations, we produce a Synthetic Digital City Dataset (SDCD) which was collected under 6 different weather driving conditions, and 6 common adverse perturbations caused by the data transmission. SDCD provides a total of 930 K high-resolution RGB images and corresponding perfect observed depth maps. The evaluation shows that depth estimation models which are trained on SDCD provide a clearer, smoother, and more precise long-range depth estimation compared to those trained on one of the best-known driving datasets KITTI. Moreover, we provide a benchmark to investigate the performance of depth estimation models in different adverse driving conditions. Instead of collecting data from the real world, we generate the SDCD under severe driving conditions with perfect observed data in the digital world, enhancing depth estimation for autonomous driving.

## Background & Summary

Depth estimation, a critical perception task in autonomous driving, aims to predict the distance and depth information of objects within a scene from images or videos^1–5^. Existing deep-learning-based depth estimation solutions have demonstrated impressive performance and potential applications^6–9^. To support the progression of these solutions, several datasets have been produced by relevant industries and research groups, which are collected from real traffic roads^10–15^. These datasets encompass traffic scenarios crucial for autonomous driving applications and generally incorporate a wealth of sensor information, including colour images from cameras and point cloud data derived from LiDAR. By interpolating sparse point clouds, the depth maps are generated, and it can provide the object-to-vehicle distance of driving scenarios to images captured by the camera, which supports deep-learning solutions for depth estimation. Several common datasets descriptions are indicated below:

### KITTI

As the best-known driving dataset which involves perception tasks in the context of autonomous driving^10^, KITTI is collected by a vehicle equipped with two high-definition colour cameras, two monochromatic cameras, a sparse Velodyne VLP-64 LiDAR scanner, and a Global Positioning System (GPS). It provides a total of 93 K RGB images and their corresponding depth maps in 56 scenes and different light conditions (Day and Night). Although the ground-truth depth maps are obtained via LIDAR in high resolution (1224 × 368), they are semi-dense and are struggling to comprehensively depict the environmental information.

### Make3D

Make3D dataset, as an early outdoor dataset that spurred the development of monocular depth estimation techniques^12^, covers 1000 outdoor scenes, including cityscapes and natural landscapes in the daytime, it consists of 400 training RGB images and 134 testing RGB images collected by the laser scanner. Although its RGB images are at high resolution (2272 × 1704), the corresponding ground-truth depth maps are provided at low resolution (305 × 55), and the different driving conditions, such as weather and light, are not comprised in.

### Scene Flow

Scene Flow dataset provides pixel-level depth and disparity information^16^, which is widely used in computer vision task research and has become one of the benchmark datasets for many vision tasks. The Scene Flow dataset comprises three distinct topics: FlyingThings3D, Driving, and Monkaa. The ‘Driving’ dataset consists of 8800 images in total, which is divided into two classes data based on different focal lengths (15 mm and 35 mm), providing valuable training data for many vision tasks (Segmentation, Optical flow, Disparity) to the driving field.

Nonetheless, the rendering quality of the driving scenes within the “Driving” dataset falls short of achieving a high level of realism. Moreover, the image content lacks the presence of pedestrians and key traffic elements such as traffic signals (including traffic lights and signal boards) that are typically found in real-world road scenes. Additionally, the dataset does not encompass common driving conditions, such as diverse weather conditions, making it unable to accurately represent the complexities of real driving scenarios.

### MegaDepth

Megadepth, as one of the largest datasets used for monocular depth estimation tasks^13^, provides a total of 130 K images. Different from other depth datasets images and depth maps captured by sensor devices, MegaDepth is composed of RGB images sourced from the internet and the corresponding ground-truth depth maps are generated via a modified algorithm based on COLMAP. Therefore, the images generated are always with noise and details missing, particularly under challenging conditions such as diverse lighting and weather conditions.

### DIODE

DIODE is the first public dataset that obtains RGBD images of indoor and outdoor scenes with one sensor suite^11^, it contains a total of 100 K diverse high-resolution (1204 × 768) RGB images with accurate, dense, far-range depth measurements. Although it covers outdoor and indoor scenes in different cities during day and night, the content of images captured consists of only static objects which unable to fully describe the information of the real world.

The existing monocular depth driving datasets play a pivotal role in the prosperity of perception algorithms but still have some key limitations. The small-scale training examples (Make3D) and low diversity of driving conditions (DIODE, Scene Flow) lead to the datasets cannot cover driving situations comprehensively in the real world, which makes it difficult to generalization to unforeseen driving conditions^17^. Moreover, the sparse and low-resolution ground-truth depth maps of datasets (KITTI, Make3D) potentially weaken the performance of depth estimation solutions, making the limited understanding of driving scenarios^4,18^.

A comprehensive dataset is thus needed to meet the requirements of driving scene diversity and the high quality of data. Therefore, to figure out the problems above, we introduce SDCD, a new synthetic dataset, which covers common driving conditions: Sunny, Rain, Snow, Sleet, Overcast and Dust. In addition, the robustness of depth estimation models is explored by considering the detrimental effects of data transmission, including Noise, Blur, Image Compression (JPEG), Colour Quantization, and Pixelation. These adverse perturbations are taken into account to assess the performance and resilience of the models. To highlight the strengths of the SDCD, a comprehensive comparison is presented in Table 1, showcasing its advantages over previous datasets.

**Table 1:** Comparisons with other depth datasets.

	KITTI	DIODE	Make3D	MegaDepth	Scene Flow	SDCD
**Size**	93 K	100 K	400	100 K	8800	930 K
**Dense Depth Map**	×	**√**	**×**	**×**	**×**	**√**
**High Resolution (1080** × **720)**	**√**	**√**	**×**	**×**	**×**	**√**
**Multiple Weather**	**×**	**×**	**×**	**×**	**×**	**√**
**Multiple Perturbations**	**×**	**√**	**×**	**×**	**×**	**√**
**Multiple Driving Scenes**	**√**	**×**	**×**	**×**	**×**	**√**

It is hard to fairly compare different datasets, but we list some characteristics here as a rough reference.

Furthermore, the adverse perturbations caused by the data transmission, such as Noise, Blur, Image quality loss during image compression (JPEG), ColourQuant, and Pixelate, are also taken into consideration to investigate the robustness of the depth estimation models. A summarized comparison (See Table 1) between SDCD and previous datasets is provided to demonstrate the advantages of SDCD.

We summarize the main contributions of this work:

- SDCD gives a large-scale, high-resolution (1080 × 720) monocular depth data, which is used to boost the development of depth estimation solutions. SDCD contains 920 K RGB images and corresponding dense depth maps with a total of 427.5 kilometers of vehicle driving mileage.
- SDCD provides adequate data collected under 6 different weather driving conditions and 6 common perturbations from the data transmission. It covers common adverse conditions in the real world to investigate the robustness of the depth estimation models. Additionally, a benchmark of depth estimating in different perturbations is provided based on the technical validation to further study the influence of the adverse disturbances on the depth estimation algorithm.
- SDCD enhances the depth observation capabilities of depth estimation models, making them more effective in real-world applications. This showcases the immense potential of synthetic datasets and the vision algorithms trained on them, particularly in autonomous driving. The validation results indicate that depth estimation models trained on the SDCD (Synthetic Driving Dataset) exhibit superior performance, with clearer, smoother, and more precise depth estimation in long-range compared to those trained on the KITTI (Real-world Driving Dataset).

## Methods

**Fig. 1:**
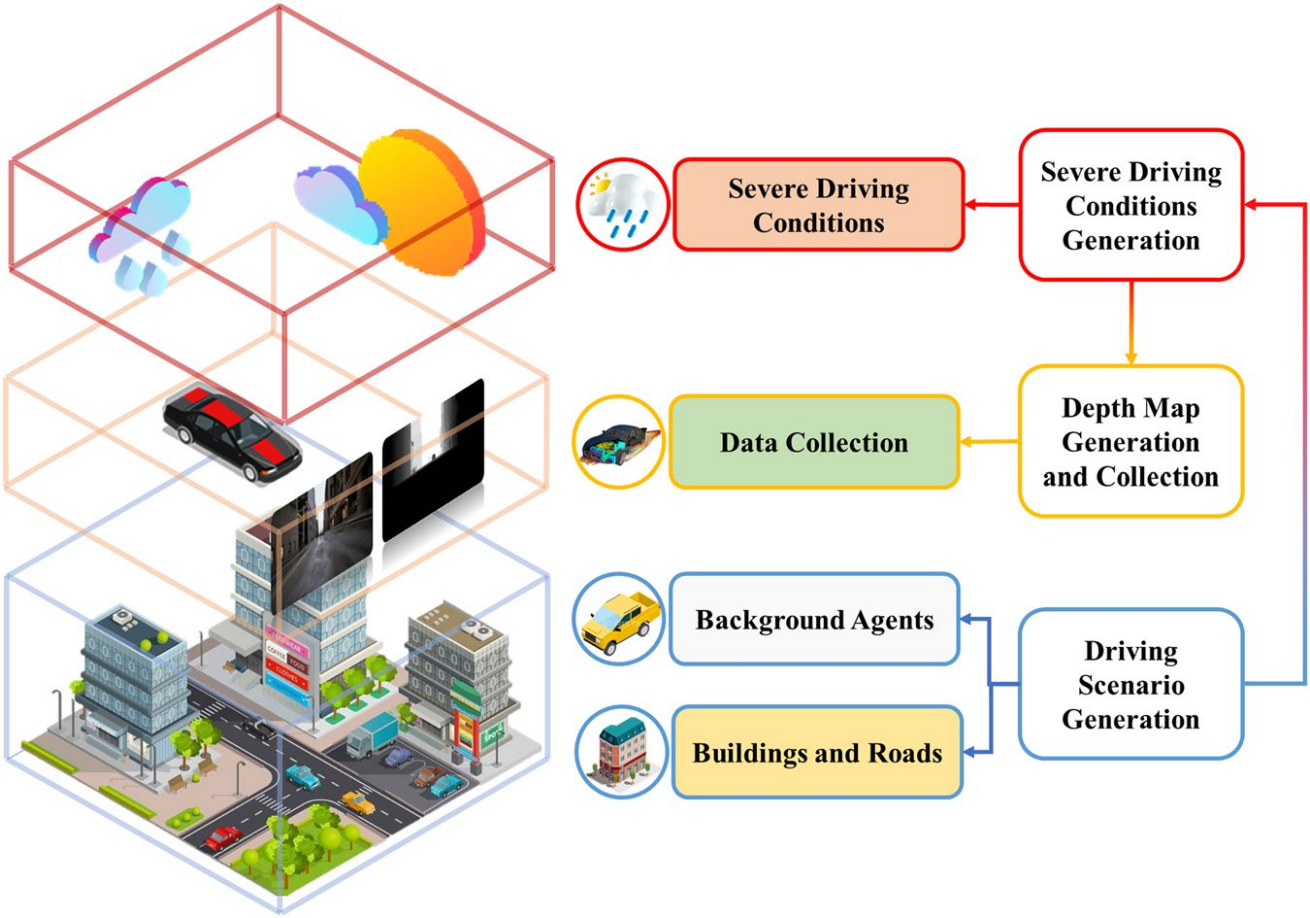
The steps of SDCD generation.

The generation of the SDCD follows three steps (See Fig. 1): a) Driving Scenario Generation, creates static city scenarios, including road maps and buildings, as well as dynamic elements like pedestrians and background vehicles. b) Severe Driving Conditions Generation, simulates different weather conditions and common perturbations from data transmission. c) Depth Map Generation and Collection, generates and collects dense, long-range ground-truth depth maps for accurate depth estimation.

### Scenario Construction

**Fig. 2:**
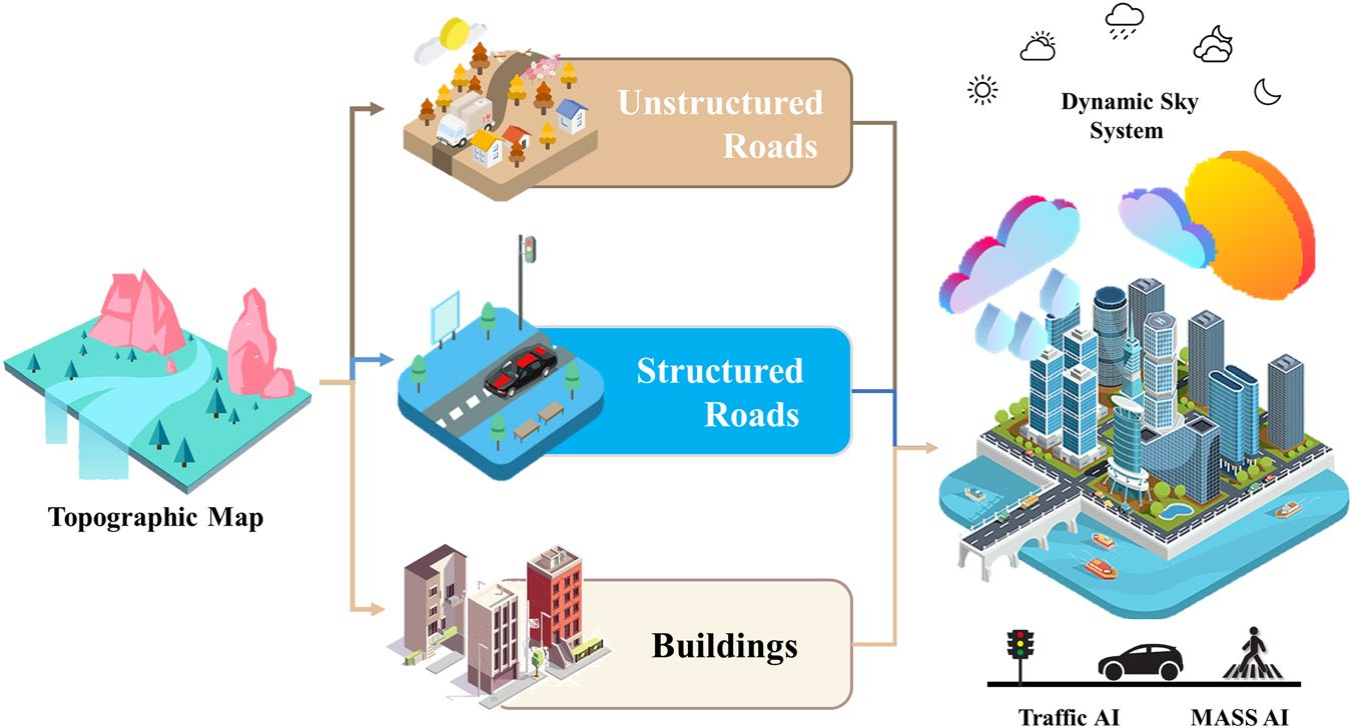
The Generation Pipeline of Digital City Driving Scenario.

The driving scenario is constructed as a modern city based on realistic rendering of Unreal Engine 5 (UE5)^18^. We designed a dedicated generation pipeline to construct the driving scenarios, which encompasses the generation of a topographic map, static scenarios, and dynamic elements, as illustrated in Fig. 2.

Topographic map as the foundation of city driving scenarios is generated based on the City Simple of UE5, covering 12 squares kilo meters. It provides significant guidance for the layout of roads and transportation networks throughout the city. Additionally, the topographic map serves as a crucial reference for accurately positioning and establishing static scenarios and dynamic elements.

Static scenarios are constructed based on the topographic map, which are mainly composed of structured roads, unstructured roads and buildings. Structured roads, such as urban freeways or arterial roads, exhibit a grid or linear layout, well-defined lane divisions and clear traffic signals. These characteristics provide geometric cues that contribute to improved accuracy in depth estimation^19^. On the contrary, unstructured roads, such as city streets and non-arterial urban roads, lack clear road demarcation and defined traffic rules, resulting in a complex road environment that poses challenges for algorithms to accurately estimate the distance and spatial relationships of objects^20^. Furthermore, buildings, serving as the fundamental elements of static scenarios, are created in diverse architectural styles and functionalities. They encompass a range of structures, including residential buildings, commercial establishments, and urban infrastructure, playing a crucial role in the generation of realistic driving scenarios.

Dynamic elements are constructed based on static scenarios, including dynamic transportation systems (traffic lights and signs), background agents (pedestrians, background vehicles), weather and day-to-night light variations. Background agents and transportation systems are randomly set into the city driving scenario, their behaviours follow the pre-set scripts which are provided by the MASS AI and Traffic AI from UE5. In addition, weather and day-to-night light variations will follow the settings of the dynamic sky system, as shown in Fig. 2.

### Perturbation Generation

Due to the fluctuations in the real-world environment and the limitation of the sensor, the driving environment data under diverse perturbed conditions cannot be perfectly observed and collected, hindering the research on the robustness of depth estimation solutions^21–27^. Benefiting from the realistic rendering capabilities of UE5, common perturbations encountered in image depth estimation tasks include: extreme weather and the perturbation generated during data acquisition and transmission, which can be effectively simulated and replicated within digital city driving scenarios^28^.

In this part, we provide a detailed introduction to the generation of common perturbations, including complex lighting conditions, rain, snow, and Gaussian noise, presented in the following manner:

### Complex Lighting Condition

In complex lighting conditions, particularly during night-time conditions, cameras will experience disturbances caused by glare and lens flares, resulting in a degradation of image quality. Specifically, the glare and lens flares give rise to complications such as blurring, scattered light, and reflections, compromising edge details of objects within the captured image.

**Fig. 3:**
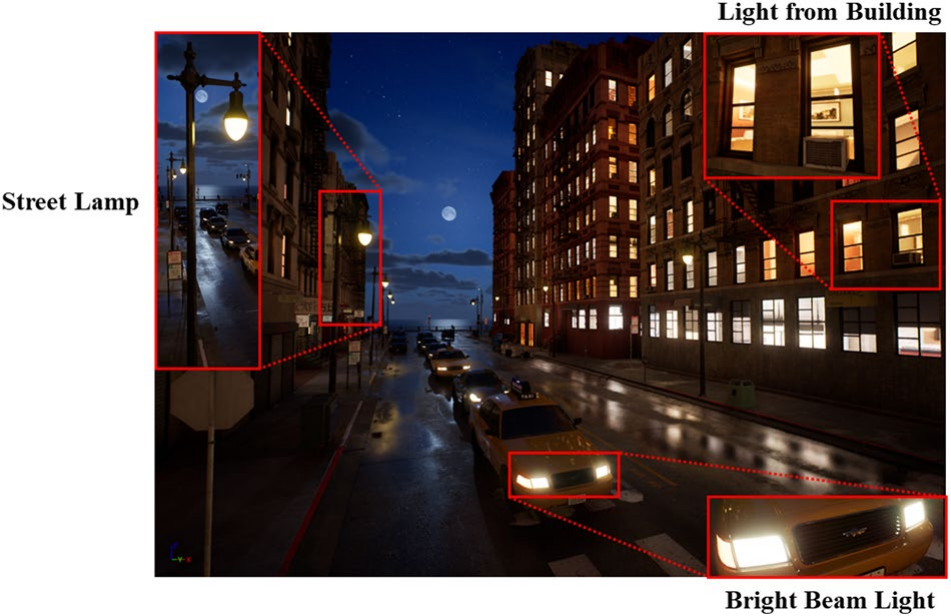
The effect drawing of city scenarios with complex light-sources.

Based on the city driving scenario above, we set up a series of light sources to create complex light conditions (See Fig. 3), such as the changing sunlight, the diverse building light, high intensity streetlamps and the bright beam light of background vehicles. Furthermore, we set a series of materials attributes of the objects in the scenarios, such as material reflection, refraction, roughness and ambient diffuse reflection, to further simulate real light variations.

### Rain

Rain, as the common weather, is always accompanied by a series of physical phenomena, such as the light scattering and occlusion effects, which severely degrades the accuracy of depth estimation solutions^29,30^. Images captured on rainy days always compose with complicate environmental information, which can be presented as^31^:

1 $$\begin{array}{c}{I}_{{\rm{rain}}}=B+{R}_{{\rm{rain}}}\end{array}$$

Where *I*_rain_ is the observed image of the monocular camera in rainy days, *B* is the clean background information of the environment, and *R*_rain_ is the rain-specific information. In general, *R*_rain_ can be divided into three different conditions:

- **Only Rainstreaks:** Rainstreaks in images can obscure scene content, reducing the visibility of the clean background and interfering with the analysis and processing conducted by depth estimation algorithms. The image captured in rainy days that only contains rainstreaks information *R*_*s*_ can be modelled as:
  2 $$\begin{array}{c}{R}_{{\rm{rain}}}=B+{R}_{s}\end{array}$$
- **Only Raindrops:** When raindrops stick to the camera lenses, the resulting images often exhibit occlusion or blurring in random small regions. These imperfections are caused by the refraction of raindrops, creating masks that obscure or distort parts of the captured scene, which can be formulated as:
  3 $$\begin{array}{c}{R}_{{\rm{rain}}}=\left(1-M\right)\odot B+{R}_{d}\end{array}$$
  Where *M* is the distortion mask, $\odot$ is the Hadamard product and *R*_*d*_ is the raindrops information.
- **Rainstreaks and Raindrops:** Rainstreaks and raindrops may appear simultaneously in the rain image observed in the real physical world. Based on the formulation above, this kind of image can be modelled as:

4 $$\begin{array}{c}{I}_{sd}=\left(1-M\right)\odot \left(B+{R}_{s}\right)+\rho {R}_{d}\end{array}$$

Where *ρ* is the global atmospheric lighting coefficient, it is notable that the fusion between rainstreaks and raindrops will change the light conditions that lead to image content distortion and then further damage image semantic information.

Based on the modeling of the image captured in rainy days, we divided the rain generation into two steps: Rainstreaks Simulation and Raindrop Simulation.

### Rainstreaks Simulation

Taking inspiration from the physics and appearance rendering of rain^32^, rainstreaks are simulated by the particle system in UE5, enabling the physical simulation of their force and movement for a realistic rainstreaks falling process. Moreover, customized materials and textures can be utilized to achieve a photorealistic appearance of rainstreaks. The specific parameters value for generating rainstreaks are outlined in Table 2.

**Table 2:** The specific parameters for generating rainstreaks.

Parameters Types	Size
**Particle Spawn Rate**	2000.0
**Rain Particle Scale**	1.0
**Rain Particle Base Colour**	Custom
**Rain Particle Alpha**	1.5
**Rain Refraction Intensity**	0.8
**Rain Ambient Light Intensity**	1.0
**Rain Particle Fall rate**	0.9

Rain Particle Base Colour is set by RGB value and other parameters are configured to maximize the simulation of rainstreaks falling in real world. The standard value unit is defined by the UE5.

The value of particle spawn rate represents the max amount of rain particles spawned per second; the rain particle fall rate is used to adjust the falling speed of rain (the bigger the faster). In order to simulate the rainfall in reality while ensuring that the scene information is not completely covered, the value of 0.9 and 2000.0 are set for particle spawn rate and rain particle fall rate respectively. In addition, to best simulate real world rain and observe the attribute of rain particles easily, the rain particle scale is set to 1.0 to simulate the real raindrop size which the diameters range is typically 1 mm^33^, and the rain particle alpha is 1.5. The base colour of rain particle is custom, the intensity of rain refraction and rain ambient light are set as 0.8 and 1.0 respectively, making the fusion between rain and the driving scenario more smoothly.

### Raindrop Simulation

**Fig. 4:**
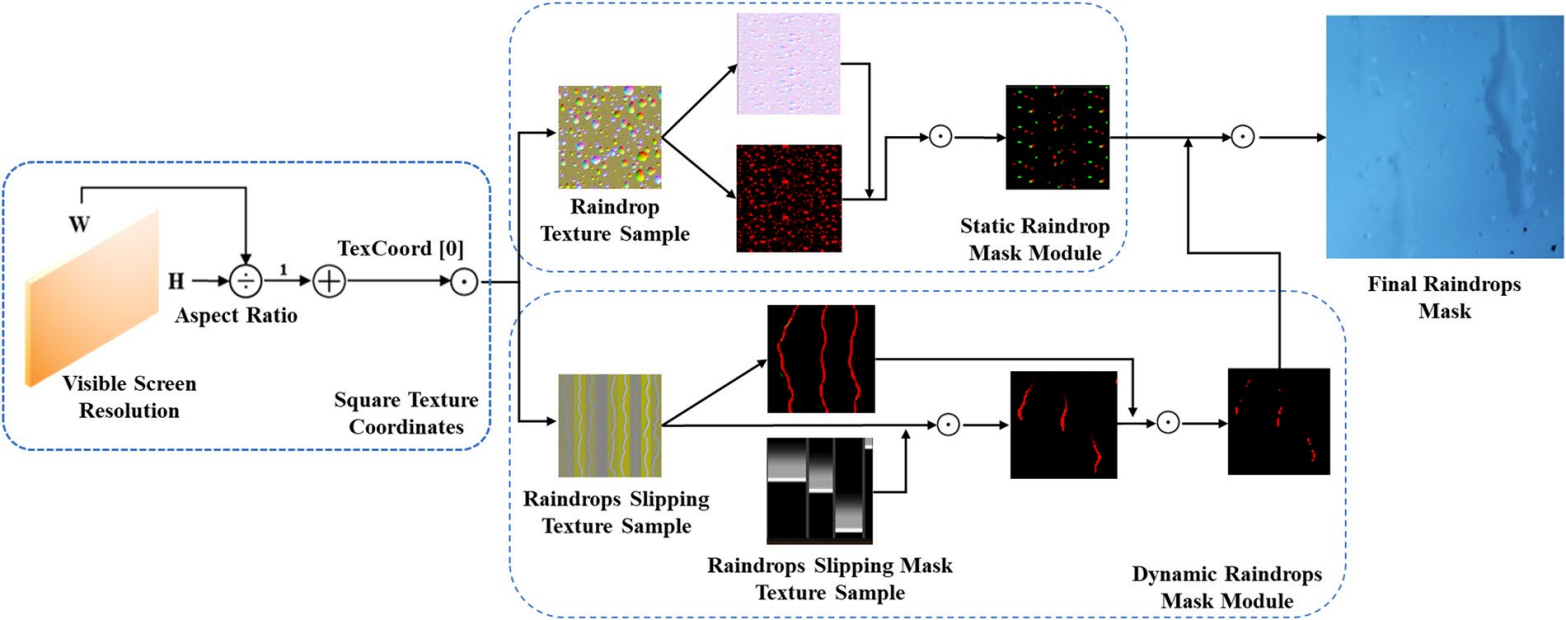
The generating process of the raindrops mask. the final raindrops mask is generated by the fusion between the result of the static raindrop mask module and the dynamic raindrops mask module. The Visible Screen Resolution, Texture Sample and TexCoord [0] are the material functional node in UE5. ⊙ is the Hadamard product.

The raindrop masks are designed to simulate the raindrops adhering to the camera lenses. We divided the raindrop mask simulation into two steps: static raindrop mask simulation and dynamic raindrop mask simulation. The detailed generation process is illustrated in Fig. 4. First, texture coordinates are acquired using the Visible Screen Resolution function and the TextCoord [0] function in UE5. Subsequently, the static raindrop mask and dynamic raindrop mask are generated separately, utilizing the corresponding normal texture and base colour texture. Finally, the raindrop mask simulation is completed by combining and blending these two masks.

### Snow

The accumulation of snow on the ground and surfaces poses challenges in extracting depth cues from texture information, leading to low accuracy and unreliable depth estimation^25,34^. Furthermore, the high albedo of snow introduces fluctuations in the intensity and colour of reflected light, resulting in an unstable performance of depth estimation^35^.

**Fig. 5:**
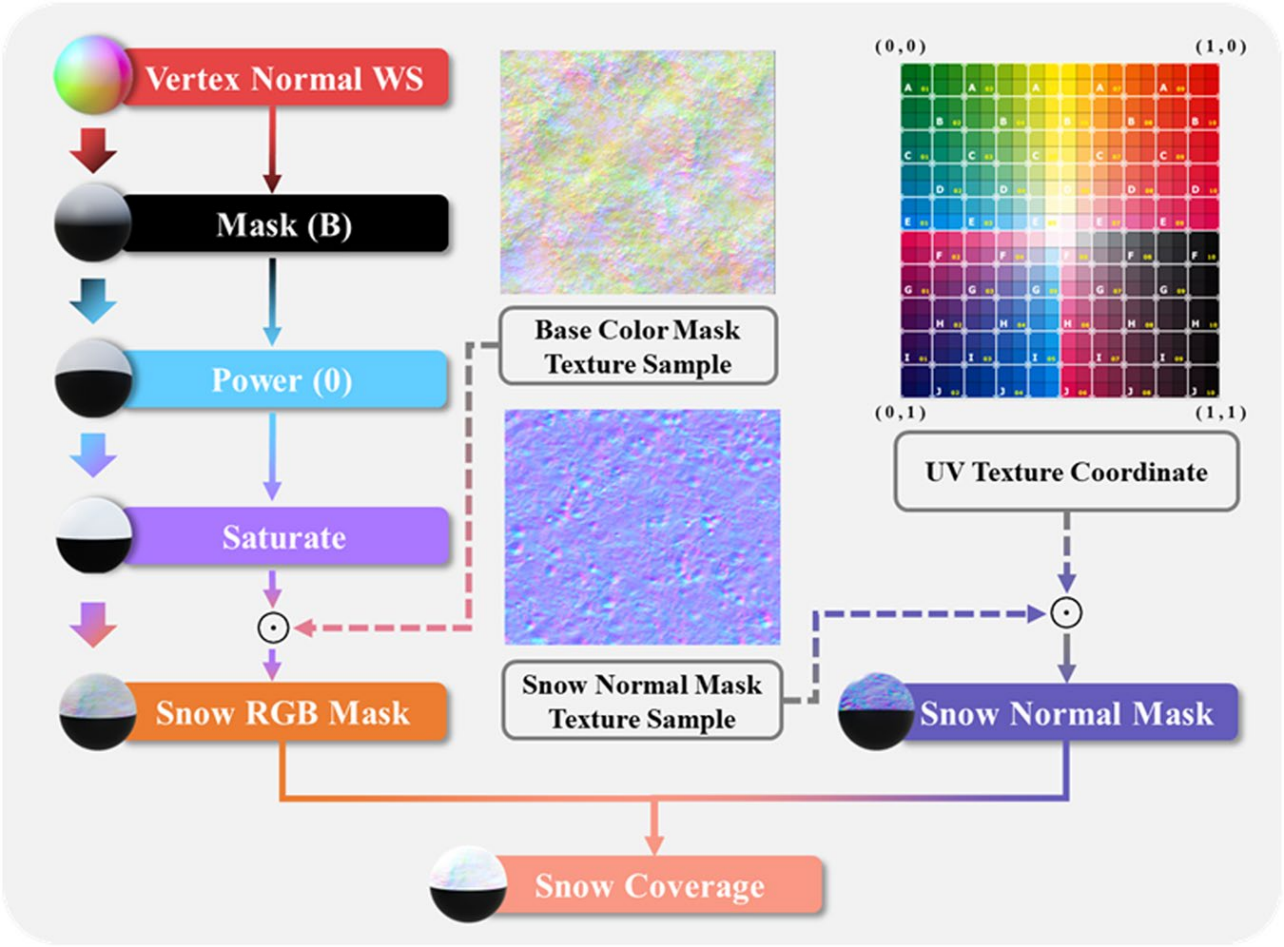
The simulation process of the Snow Coverage. The material sphere on the left of each module is the corresponding visual effect, ⊙ is the Hadamard product.

We divided snow simulation into two parts: the snow coverage simulation and the snow particle simulation. The specific generation of snow coverage is shown in Fig. 5. First, the vertex object surface is obtained by the Vertex normal WS function and Mask (B) function, then the height of snow coverage and the contrast between snow and scene object are generated based on Power (0) function and Saturate function, at last, the snow RGB Mask is obtained via the Saturate function result and the texture sample of snow base colour. Additionally, the snow normal mask is generated based on the fusion between the texture sample of snow normal and the UV texture coordinate. Finally, the simulation of the snow coverage effect is completed by the combination of the snow RGB mask and snow normal mask.

Following similar simulation procedures as rainstreak particles. Snowflakes are simulated using the particle system in UE5 as well, the parameters of snow particle simulation are shown in Table 3. The number of particle spawn rate represents the max amount of snowflakes particles spawned per second; the snowflakes particle fall rate is used to adjust the falling speed of snow (the smaller the slower). In order to simulate the snow in reality while ensuring that the scene information is not completely covered, the value of 0.25 and 1800.0 are set for particle spawn rate and snow particle fall rate respectively. As the opacity of snowflakes is higher than rain particles and the snowflakes is earlier than rain particle to be observed, the snowflakes alpha is smaller than rain particle alpha which is 0.43. In addition, to best simulate real world snow, the snowflakes scale is set to 2.25 to simulate the real snow size which the diameters range is typically 2.25 mm^36^. The base colour and normal of snowflakes are custom, the snow ambient light is 1.5 which is higher than rain particles, as the high albedo of snow.

**Table 3:** The specific parameters for generating snowflakes.

Parameters Types	Size
**Particle Spawn Rate**	1800.0
**Snowflakes Scale**	2.25
**Snowflakes Base Colour**	Custom
**Snowflakes Normal**	Custom
**Snowflakes Alpha**	0.43
**Snow Ambient Light Intensity**	1.5
**Snow Particle Fall rate**	0.25

Snowflakes base colour and Snowflakes normal are set by custom texture sample and other parameters are configured to maximize the simulation of snow falling in real world. The standard value unit is defined by the UE5.

### Gaussian Noise

During the process of capturing, encoding, and transmitting images, noise can be introduced from various sources including sensor devices, thermal effects, signal interference, compression artifacts, and challenging lighting conditions, affecting image quality and clarity^37^. As it is difficult for depth estimation models that extract reliable features from noise images, the image with noise will adversely affect the quality of images. which can be formulated as:

5 $$\begin{array}{c}A\left(x,y\right)=H\left(x,y\right)+B\left(x,y\right)\end{array}$$

where *x*,*y* are the coordinates of the pixel, *A*(*x*,*y*) represent the noisy image, *H*(*x*,*y*) represent the noise information of each pixel and *B*(*x*,*y*) is the information of each pixel from the original image.

**Fig. 6:**
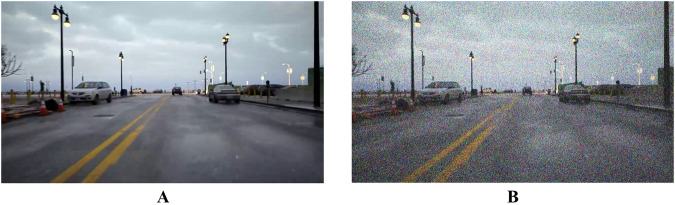
The original image and corresponding Gaussian noisy image. A is the original image, B is the corresponding Gaussian noisy image.

The widespread utilization and favourable mathematical properties of Gaussian noise make it a common choice for modeling randomness or uncertainty, allowing for the simulation of real-world noise^38^. In order to generate the device communication perturbation during driving, we imposed the Gaussian noise on the RGB image, it can cause the loss of fine details in the image, making it difficult for the depth estimation algorithm to accurately determine the distance of objects in the scene. The Gaussian Probability Density Function *p*(*z*) is:

6 $$\begin{array}{c}p\left({\rm{z}}\right)=\frac{1}{\sqrt{2\pi }\sigma }{e}^{\frac{-{(z-\mu )}^{2}}{2{\sigma }^{2}}}\end{array}$$

where *z* represents the gray value of the image pixel, *μ* represents the average value of the pixel value, and *σ* represents the standard deviation of the pixel value. we assign each pixel a random probability value from a standard Gaussian distribution $N\left(\mu ,{\sigma }^{2}\right)$ and impose it on the RGB images. The noisy image is shown in Fig. 6.

### Ground-truth Depth Map Generation

**Fig. 7:**
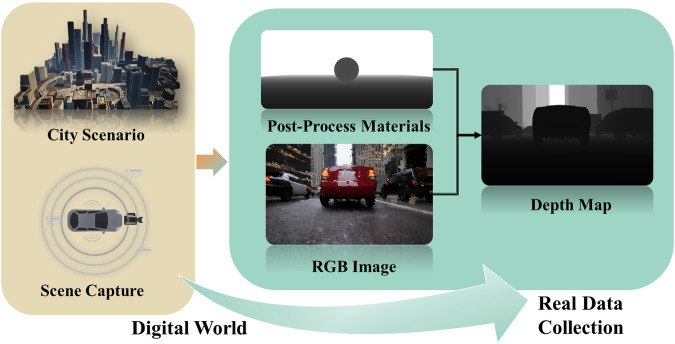
The Schematic Diagram of Ground-truth Depth Map Generation.

Different from the sparse depth map collected from the LiDAR which is difficult to fully demonstrate the environmental information. We complete the information migration from the digital world to the real data collection via UE5 (Fig. 7). First, the whole digital world is obtained by the combination between city scenario and scene capture component, then the depth map is generated based on the combination of Post-Process material and RGB image which captured from the digital world.

## Data Records

**Fig. 8:**
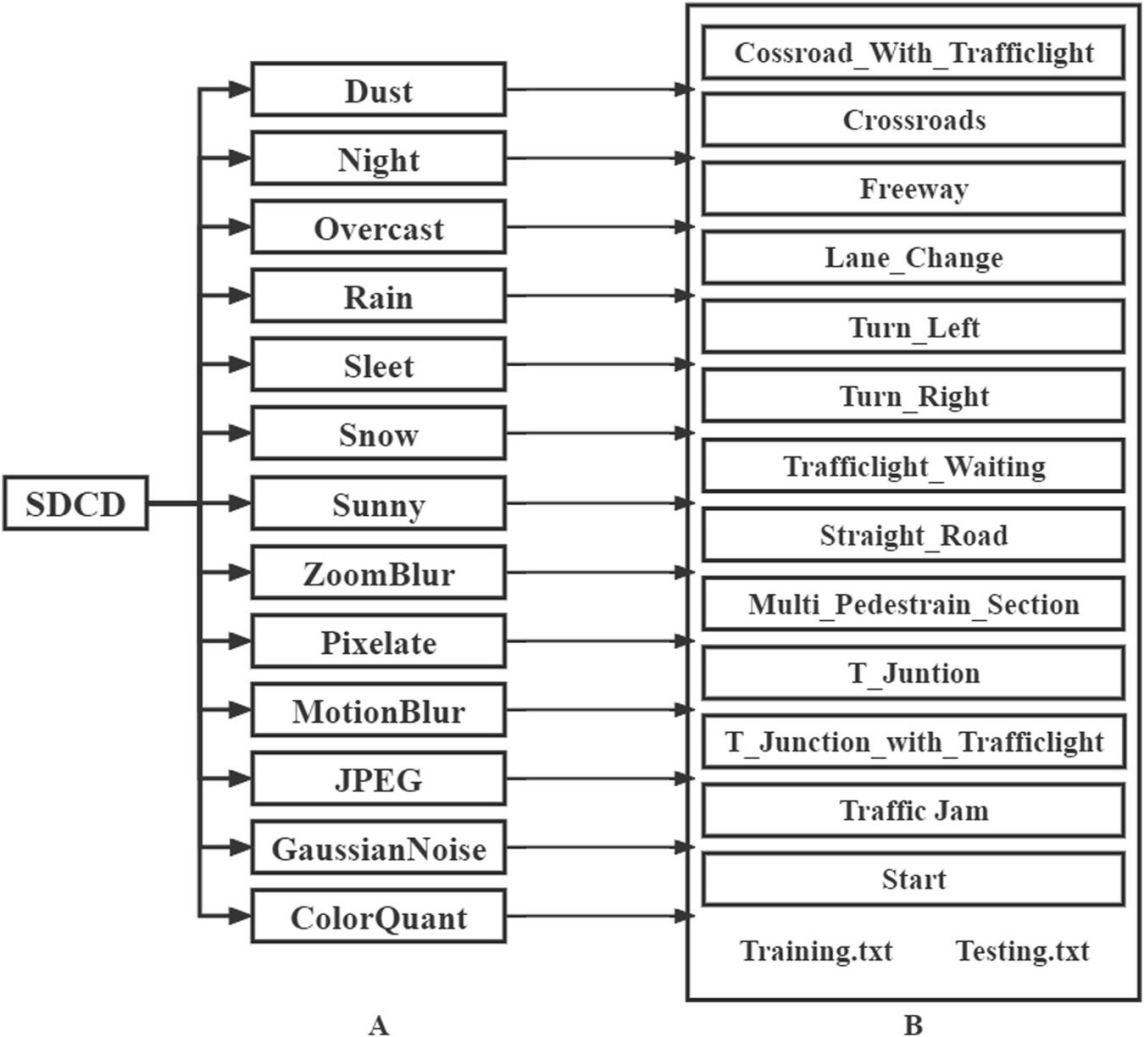
The data directory hierarchy and content of SDCD. The directory hierarchy have two levels, level A consists of 13 folders about driving conditions, level B consists of 13 folders about driving scenes and two txt file about the location index of each training and testing data. Each folder in level A has its own level B folders.

SDCD is available under ScienceDB repositories^39^ in zip compression format. In order to better use the dataset for depth estimation tasks under different conditions, we divide SDCD into several folders according to the type of perturbation with the RGB images and the corresponding ground-truth depth maps, each folder stored with the data file and two txt files (the location index of each training and testing data). The data directory hierarchy and content are shown in Fig. 8.

## Technical Validation

To investigate the reliability of the depth estimation models trained on the SDCD in the real-world applications, we adopt two the-state-of-the-art estimation solutions (BTS^40^ and NeWCRFS^41^) on KITTI and SDCD respectively, which hyperparameters are shown in Table 4. To validate the performance of these two models trained under SDCD and KITTI, we obtain a series of city driving videos from the YouTube (https://www.youtube.com/@jutah), a total of 2000 images were extracted from these videos every 10 frames to create the evaluation dataset. Images of the evaluation dataset are not in both KITTI and the proposed SDCD datasets, all of them are high resolution (1280×720), the content of images are daily city traffic scenario which can highly reflect the reliability of SDCD in real-world applications.

**Table 4:** The hyperparameters of BTS model and NeWCRFS model.

	BTS	NeWCRFS
**Batch Size**	4	5
**Epochs**	1000	1000
**Learning Rate**	1e-4	2e-5
**Weight Decay**	1e-2	1e-2
**Adam Eps**	1e-3	1e-3

These hyperparameters of each model are same in training on both KITTI and SDCD.

**Fig. 9:**
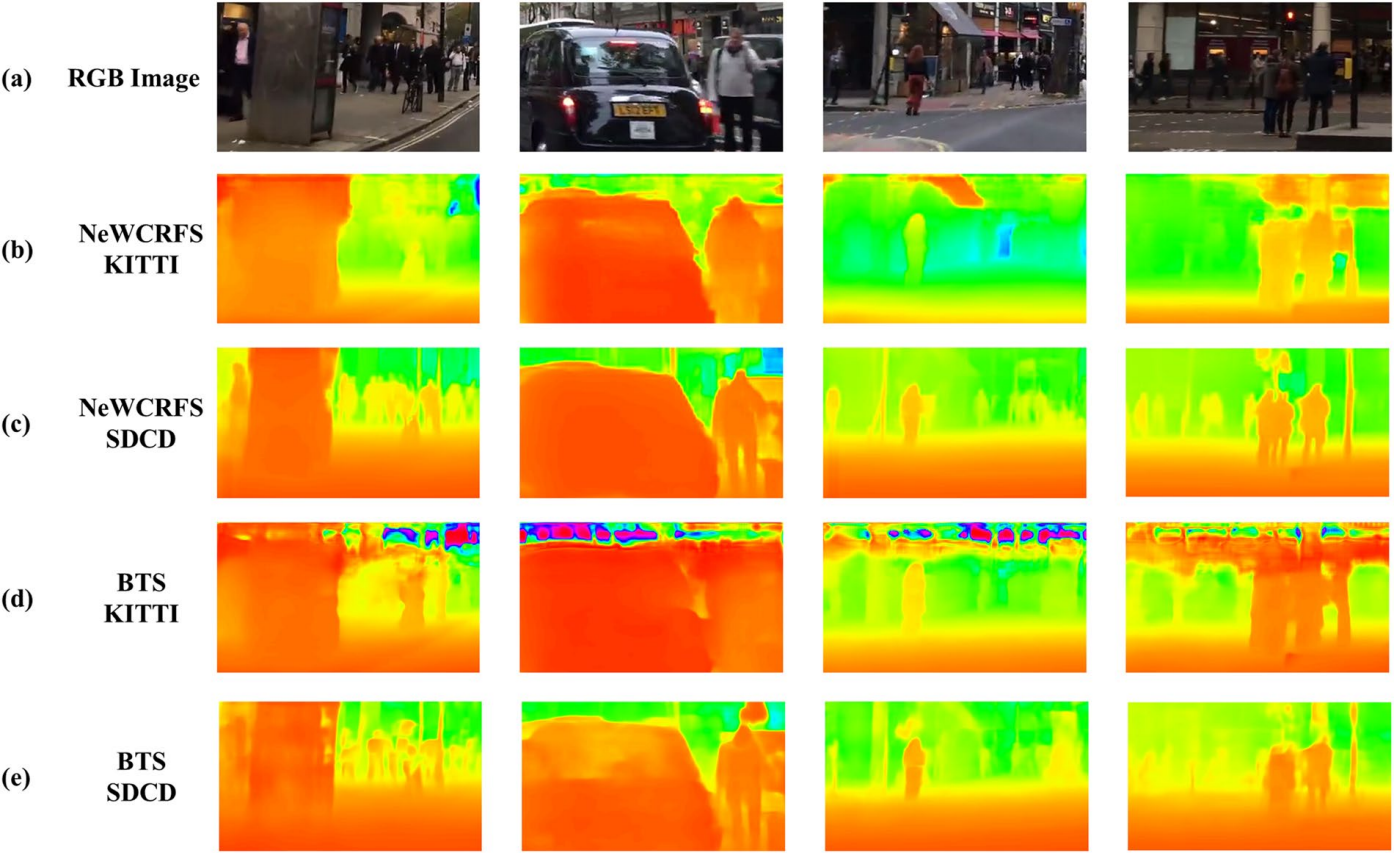
The performance comparison of depth estimation models (BTS, NeWCRFs) which were adopted on KITTI and SDCD respectively. RGB Image row are the images that not belong to the KITTI or SDCD. (**a**) is the RGB images that not in KITTI or SDCD, (**b**) is the depth estimation result from NewCRFS that adopted on KITTI, (**c**) is the depth estimation result from NeWCRFs that adopted on SDCD, (**d**) is the depth estimation result from BTS that adopted on KITTI, (**e**) is the depth estimation result from BTS that adopted on SDCD. The mosaic regions in RGB images are used to protect people’s privacy which is not impacting the training performance.

The results from the validation dataset demonstrate that models (BTS and NeWCRFS) trained on the SDCD have better performance of depth estimation from single image than that trained on the KITTI (See Fig. 9), they are able to estimate scene information at further distances. Moreover, models trained on SDCD have a clearer features estimation of scene object in the near distance than models trained on KITTI.

To further investigate the specific impact on depth estimation performance under different driving conditions and perturbations. We train BTS and NeWCRFs on the corresponding training data types of SDCD, then assess the results with evaluation metrics which include Scale Invariant Logarithmic Error (SILog), Absolute Relative Error (Abs Rel), Square Relative Error (Sq Rel), Root Mean Square Error (RMSE), and the Logarithm Root Mean Square Error (log RMS)^42^. These metrics are formulated as:

7 $${\rm{SI}}\,{\rm{log\; =}}\frac{1}{n}\sum _{i}{{\left({y}_{i}-{y}_{i}^{* }\right)}^{2}-\frac{1}{{n}^{2}}\left(\sum _{i}\left(\log {y}_{i}-\log {y}_{i}^{* }\right)\right)}^{2}$$

8 $$\begin{array}{c}{\rm{Abs}}\,{\rm{Rel}}=\frac{1}{T}\sum _{i\in T}\frac{{y}_{i}-{y}_{i}^{* }}{{y}_{i}^{* }}\end{array}$$

9 $$\begin{array}{c}{\rm{Sq}}\,{\rm{Rel}}=\frac{1}{T}\sum _{i\in T}\frac{{\left\Vert {y}_{i}-{y}_{i}^{* }\right\Vert }^{2}}{{y}_{i}^{* }}\end{array}$$

10 $$\begin{array}{c}{\rm{RMS}}=\sqrt{\frac{1}{T}\sum _{i\in T}{\left\Vert {y}_{i}-{y}_{i}^{* }\right\Vert }^{2}}\end{array}$$

11 $$\begin{array}{c}{\rm{Log}}\,{\rm{RMS}}=\sqrt{\frac{1}{\left|T\right|}\sum _{i\in T}{\left\Vert \log {y}_{i}-\log {y}_{i}^{* }\right\Vert }^{2}}\end{array}$$

Where *y*_*i*_ and ${y}_{i}^{* }$ are the predicted depth and ground truth depth respectively at the pixel indexed by *i*, *T* is the total number of pixels in all the evaluated images.

**Fig. 10:**
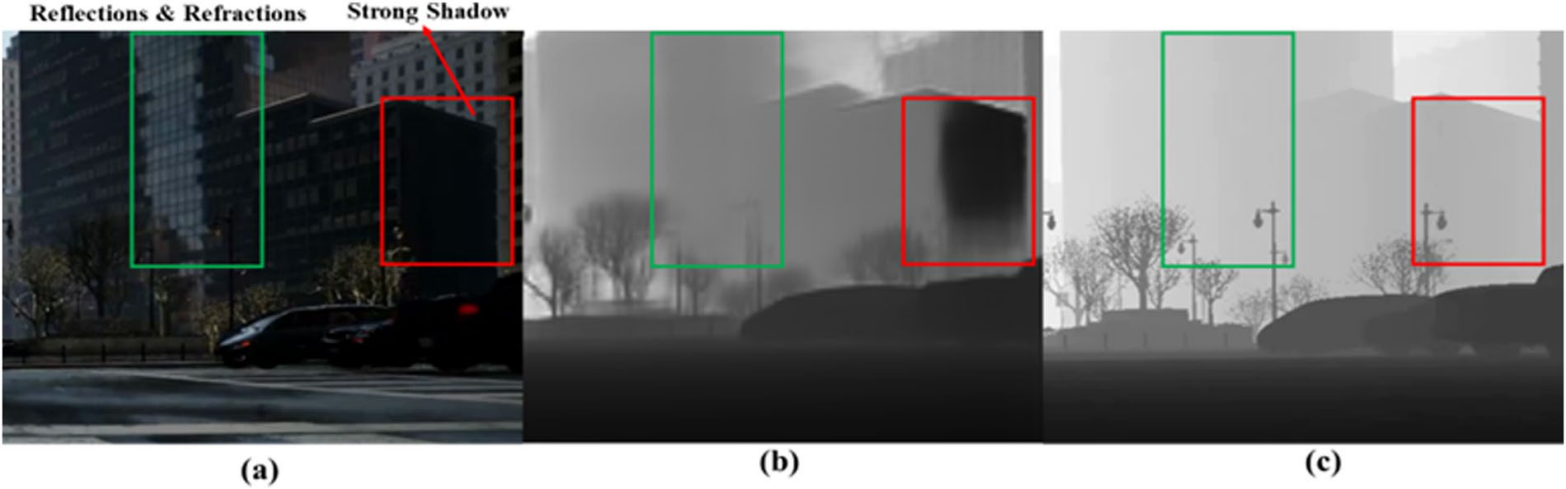
The specific effect of shadow variations, reflections and refractions on depth estimation under sunny/normal driving conditions. (**a**) is the RGB image from the sunny / normal driving condition in proposed SDCD. (**b**) is the depth estimation result. (**c**) is the Ground-truth depth map of proposed SDCD. Green box is the reflection and refraction region, red box is the strong shadow region.

The experimental results (see Tables 5, 6) provide a benchmark for depth estimation performance. It is notable that both BTS and NeWCRFs have generally good depth estimation performance under sunny / normal driving conditions. However, some depth estimation results from these two models on sunny / normal type achieve lower performance than that on other types, after analysing the results, we figure out the main causes are shadow variations, reflections and refractions (see Fig. 10).

**Table 5:** The benchmark of depth estimation performance from BTS model.

BTS									
	AbsRel	SqRel	RMSE	RMSElog	SILog	d1	d2	d3	Types
**Driving Conditions↓**	**0.062**	**0.012**	**0.007**	**0.262**	**0.254**	**0.958**	**0.981**	**0.988**	**Sunny**
	0.373	0.08	0.012	0.384	0.291	0.557	0.819	0.909	Snow
	0.265	0.077	0.014	0.342	0.199	0.633	0.769	0.849	Rain
	0.271	0.057	0.015	0.471	0.273	0.382	0.619	0.907	Overcast
	0.269	0.104	0.015	0.358	0.347	0.705	0.911	0.95	Sleet
	0.313	0.164	0.039	0.668	0.473	0.491	0.596	0.681	Night
	0.760	0.408	0.054	2.274	0.951	0.045	0.106	0.188	Dust
**Perturbation Types↓**	**0.062**	**0.012**	**0.007**	**0.262**	**0.254**	**0.958**	**0.981**	**0.988**	**Normal**
	0.267	0.054	0.015	0.238	0.147	0.386	0.670	0.895	Pixelate
	0.283	0.064	0.017	0.529	0.325	0.387	0.610	0.868	ColourQuant
	0.309	0.079	0.018	0.512	0.370	0.411	0.679	0.860	JPEG
	0.315	0.070	0.019	0.589	0.357	0.355	0.538	0.768	ZoomBlur
	0.357	0.090	0.020	0.761	0.456	0.361	0.516	0.696	MotionBlur
	0.923	0.304	0.040	0.917	0.842	0.097	0.207	0.329	GaussianNoise

**Table 6:** The benchmark of depth estimation performance from NewCRFs model.

NewCRFs									
	AbsRel	SqRel	RMSE	RMSElog	SILog	d1	d2	d3	Types
**Driving Conditions↓**	0.061	0.002	0.030	0.119	0.116	0.949	0.984	0.992	**Sunny**
	0.36	0.072	0.011	0.436	0.353	0.562	0.814	0.909	Snow
	0.099	0.017	0.011	0.197	0.178	0.918	0.965	0.982	Rain
	0.271	0.1	0.017	0.631	0.626	0.674	0.894	0.944	Overcast
	0.259	0.057	0.018	0.71	0.554	0.386	0.662	0.922	Sleet
	0.576	0.26	0.027	0.85	0.667	0.414	0.572	0.718	Night
	0.796	0.425	0.055	2.348	0.906	0.035	0.075	0.128	Dust
**Image Types↓**	0.061	0.002	0.030	0.119	0.116	0.949	0.984	0.992	**Normal**
	0.263	0.061	0.019	0.706	0.542	0.416	0.665	0.901	Pixelate
	0.265	0.061	0.019	0.766	0.621	0.397	0.675	0.901	ColourQuant
	0.311	0.071	0.019	0.762	0.542	0.363	0.545	0.784	JPEG
	0.316	0.082	0.022	0.777	0.556	0.336	0.536	0.786	ZoomBlur
	0.457	0.142	0.026	1.207	0.756	0.317	0.389	0.519	MotionBlur
	0.474	0.156	0.026	1.167	0.576	0.336	0.431	0.52	GaussianNoise

### Shadow Variations

The light in sunny day can cause strong shadows. Depth estimation in these shadow regions may be affected by lighting changes, resulting in inaccurate depth estimation results.

### Strong Shadow, Reflections and Refractions

Driving environments in sunny day may have more ambient reflections and refractions that can complicate depth estimation process. Especially in the case of transparent and reflective materials, the depth estimation algorithm may be disturbed.

## Usage Notes

All data are published in image and txt file format, users can access them entirely without any further permission. SDCD covers the different weather driving conditions and diverse image perturbations in autonomous perception tasks. By providing the RGB images and corresponding ground-truth depth maps, we hope that SDCD can be used as a training dataset to improve the performance and robustness of depth estimation algorithms in autonomous application.

As the dataset is quite large, we divide SDCD into 13 folders based on the training data type in order to help users use it according to their computing conditions, also we provide corresponding tutorial of how to use this data on GitHub website.

### Supplementary information

Table1

Table5

Table6

## Data Availability

A git repository is publicly available at https://github.com/ReparkHjc/SDCD, in this repository several python scripts for visualisation, benchmarking and data pre-processing are available.
